# McDonald Criteria 2010 and 2005 Compared: Persistence of High Oligoclonal Band Prevalence Despite Almost Doubled Diagnostic Sensitivity

**DOI:** 10.3390/ijms17091592

**Published:** 2016-09-21

**Authors:** Philipp Schwenkenbecher, Anastasia Sarikidi, Ulrich Wurster, Paul Bronzlik, Kurt-Wolfram Sühs, Peter Raab, Martin Stangel, Refik Pul, Thomas Skripuletz

**Affiliations:** 1 Clinical Neuroimmunology and Neurochemistry, Department of Neurology, Hannover Medical School, Hannover 30625, Germany; schwenkenbecher.philipp@mh-hannover.de (P.S.); sarikidi.anastasia@mh-hannover.de (A.S.); wurster.ulrich@mh-hannover.de (U.W.); suehs.kurt-wolfram@mh-hannover.de (K.-W.S.); stangel.martin@mh-hannover.de (M.S.); pul.refik@mh-hannover.de (R.P.); 2 Department of Diagnostic and Interventional Neuroradiology, Hannover Medical School, Hannover 30625, Germany; bronzlik.paul@mh-hannover.de (P.B.); raab.peter@mh-hannover.de (P.R.)

**Keywords:** multiple sclerosis, OCB, CSF

## Abstract

The 2010 McDonald criteria were developed to allow a more rapid diagnosis of relapsing-remitting multiple sclerosis (MS) by only one MRI of the brain. Although cerebrospinal fluid (CSF) is not a mandatory part of the latest criteria, the evidence of an intrathecal humoral immunoreaction in the form of oligoclonal bands (OCB) is crucial in the diagnostic workup. To date, the impact of the 2010 McDonald criteria on the prevalence of OCB has not been investigated. We retrospectively evaluated data of 325 patients with a clinical relapse suggestive of demyelination that were treated in a German university hospital between 2010 and 2015. One hundred thirty-six patients (42%) were diagnosed with MS and 189 patients with CIS when the criteria of 2010 were applied. The criteria of 2005 allowed only 70 patients (22%) to be designated as MS. In contrast, the prevalence of OCB was marginal affected in MS patients with 96% for the criteria of 2010 and 98.5% for the criteria of 2005. In conclusion, OCB are prevalent in most MS patients and reflect the chronic inflammatory nature of the disease. We recommend CSF examination to exclude alternative diagnoses and reevaluation of the diagnosis MS in patients with negative OCB.

## 1. Introduction

Approximately 85% of patients with multiple sclerosis (MS) initially present with a first relapse-like episode of neurological symptoms. When dissemination in space and time are not evident such an event is called clinically isolated syndrome (CIS) [1,2]. Quick and reliable diagnosis is required since an early start of a disease-modifying immunomodulatory treatment in patients with MS is considered to exert a beneficial impact on the disease course [3,4,5,6]. The analysis of cerebrospinal fluid (CSF) is not only a crucial diagnostic tool to exclude other causes mimicking MS but also a source of possible biomarkers [7,8].

Intrathecal synthesis of IgG antibodies in the form of oligoclonal bands (OCB) occurs in both, patients with MS and CIS [9]. The presence of OCB in patients with CIS is considered to be a prognostic marker for developing MS [10,11]. The correlation between the OCB status in MS patients and the disease course is controversially discussed. Some studies reported that MS patients without OCB have a slower disease progression, and thus, a better long-term prognosis [12].

A large meta-analysis including more than 12,000 participants showed that the detection of OCB has a diagnostic sensitivity of approximately 88% and a specificity of at least 86% in MS patients [13,14]. However, there is a lack of data regarding the OCB status in patients that were diagnosed with MS according to the last revision of the McDonald criteria in 2010. Those new criteria allow the diagnosis of MS at an earlier stage already after the first clinical event when dissemination of inflammatory lesions in space and time can be demonstrated by brain MRI [15].

Although CSF diagnostics are no longer a mandatory part of this new criteria, the detection of OCB provides evidence for the inflammatory nature of the disease, has prognostic significance and is important to exclude alternative diagnoses. The aim of this study was to determine the prevalence of OCB in patients diagnosed with MS according to the McDonald criteria of 2010.

## 2. Results

A total number of 325 patients were included in the study (Table 1). 136 patients (42%) were diagnosed with MS according to the revised McDonald criteria of 2010. MS was diagnosed with one relapse due to evidence of dissemination in space and time demonstrated by MRI in 60 of these patients (44%). The other 76 patients with newly diagnosed MS (56%) reported one or more previous relapses fulfilling dissemination in time. MRI identified dissemination in space in all of these 76 patients while dissemination in time (contrast enhanced lesions) was found in 39 patients.

The remaining 189 patients were diagnosed with CIS. MRI showed abnormalities in 131 of the CIS patients (69%). Dissemination in space was found in 92 patients (49%), while 39 patients had lesions in one region only. Symptomatic contrast enhanced lesions were detected in 16 patients (all located in the spinal cord). Fifty-eight patients (31%) showed normal baseline MRI.

### 2.1. Clinical Characteristics in Patients with MS and CIS

In patients with MS (McDonald criteria 2010), the median age was 31 years (range 17–73 years) and the female sex was predominant with 70%. Optic neuritis was the most frequent clinical presentation (32%), followed by spinal cord symptoms (22%), paresis/sensory symptoms suggesting cerebral lesions (22%), brainstem symptoms (14%), and a polysymptomatic presentation (10%; Table 1).

The median age of patients with CIS was 34 years (range 16–73 years) and 63% of these patients were female. The majority of patients with CIS presented with symptoms of optic neuritis (72%) followed by spinal cord symptoms (14%), paresis/sensory symptoms suggesting cerebral lesions (6%), and brainstem symptoms (8%).

### 2.2. CSF Changes in MS Patients

**Figure 1:**
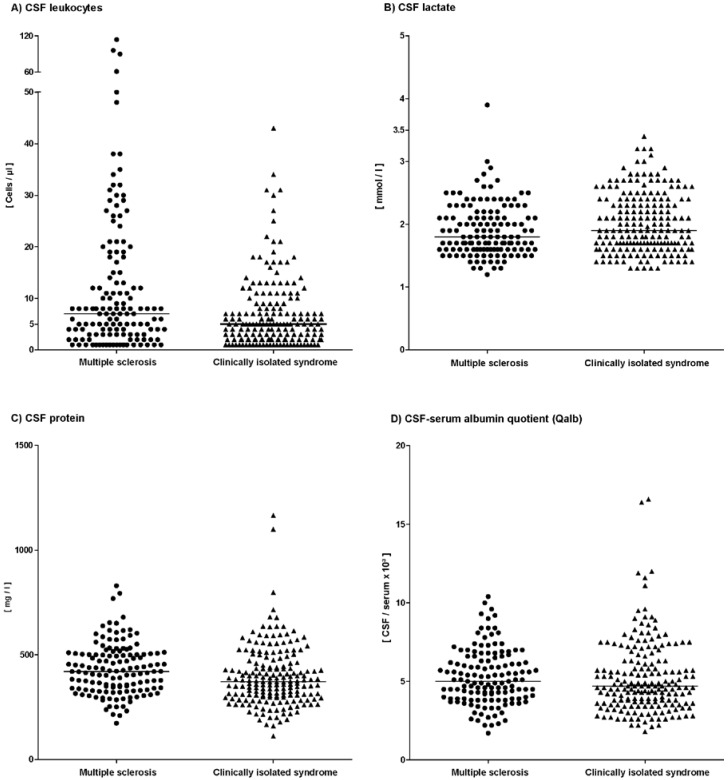
Cerebrospinal fluid results in patients with multiple sclerosis and clinically isolated syndrome according to the McDonald criteria 2010. Graphs show the distribution of cell count (**A**), lactate (**B**), total protein (**C**), and albumin CSF/serum quotients (**D**). Bars represent median values in each group.

Eighty-nine patients (65%) with MS (McDonald criteria 2010) exhibited a slightly increased CSF cell count (median 7 cells/µL, range 1–114 cells/µL; Figure 1, Table 1). Except for five patients, cell count was lower than 50 cells/µL. Two of these five patients presented with myelitis, two with optic neuritis and one with a brainstem lesion. The median level lactate amounted to 1.8 mmol/L (range 1.2–3.9 mmol/L). Only one patient with a brainstem lesion exhibited a pathologically increased lactate concentration of 3.9 mmol/L.

CSF total protein was elevated in 38 patients (median 421 mg/L, range 173–830 mg/L). Measurements of QAlb, which is the best indicator for a blood-CSF barrier dysfunction, revealed age-corrected increased values in 36 patients (median 5.0, range 1.7–10.4). Barrier impairment was only mild in all of these patients (QAlb < 15).

OCB restricted to the CSF were found in all but five patients (96%) indicating intrathecal IgG synthesis. Ten of these patients showed a combination of OCB exclusively in the CSF plus identical OCB in CSF and serum (type 3). Only one patient showed a weak OCB pattern with only three CSF bands.

Quantitative (Reiber-Felgenhauer graphs) intrathecal synthesis of immunoglobulins of either IgM, or IgG, or IgA occurred in 96 patients (71%). Intrathecal synthesis of IgG was found in 85 patients (63%), IgM synthesis was found in 48 patients (35%), and IgA synthesis was found in 18 patients (13%).

A combined three-class reaction of intrathecal synthesis of IgG, IgM, and IgA was found in 10 patients (7%; Table 2). In addition, two-class reactions with the following combinations were found: IgG + IgM in 29 patients (21%), IgG + IgA in 5 patients (4%), and IgM + IgA in 1 patient (1%). Isolated IgG synthesis was found in 39 patients (29%), isolated IgM was found in 7 patients (5%), and isolated IgA synthesis was found in 2 patients (1%).

When OCB were included as a sign of intrathecal IgG synthesis (qualitative method) a combined three-class reaction of immunoglobulins was then found in 11 patients (8%; Table 2). The two-class reactions of IgG + IgM in 37 patients (27%) and IgA + IgG in 7 patients (5%) were found. Isolated IgG synthesis occurred in 73 patients (54%). A two-class reaction of IgM + IgA as well as isolated IgM and isolated IgA synthesis was not found.

### 2.3. CSF Changes in CIS Patients

Ninety-eight patients (52%) with CIS presented a slightly increased CSF cell count (median 5 cells/µL, range 1–43 cells/µL; Figure 1, Table 1). For CSF lactate concentration a median level of 1.9 mmol/L was found (range 1.3–3.4 mmol/L). CSF total protein was increased in 40 patients (median 372 mg/L, range 112–1166 mg/L). A blood-CSF barrier dysfunction was found in 42 patients (median 4.7, range 1.8–16.6). Barrier impairment was mild in 40 of these patients (Qalb < 15) and moderate in two patients. One patient with moderate barrier impairment had undergone neurosurgery for a herniated spinal disc two months ago while the other patient displayed degenerative cervical spine abnormalities.

OCB restricted to the CSF were found in 104 patients (55%). Nine of these patients showed a combination of OCB restricted to the CSF and identical oligoclonal IgG bands in CSF and serum (type 3). Five patients with CSF OCB showed weak values with two or three CSF bands (type 2a or 3a).

Quantitative intrathecal synthesis of immunoglobulins of either IgM, IgG, or IgA occurred in 64 patients (34%). Intrathecal synthesis of IgG was found in 62 patients (33%), IgM synthesis was found in 27 patients (14%), and IgA synthesis was found in 7 patients (4%).

A combined three-class reaction of intrathecal synthesis of IgG, IgM, and IgA was found in 5 patients (3%; Table 2). Two-class reactions with the following combinations were found: IgG + IgM in 20 patients (11%), IgG + IgA in 2 patients (1%), and IgM + IgA in 1 patient (1%). Isolated IgG synthesis was found in 36 patients (19%) and isolated IgM was found in 1 patient (1%). Isolated IgA synthesis did not occur.

When OCB were included as a sign of intrathecal IgG synthesis (qualitative method) a combined three-class reaction of immunoglobulins was then found in 6 patients (3%). The two-class reactions of IgG + IgM in 21 patients (11%) and IgG + IgA in 2 patients (1%) were found. Isolated IgG synthesis occurred in 77 patients (41%). A two-class reaction of IgM + IgA as well as isolated IgM and isolated IgA synthesis was not found.

### 2.4. Comparison Between the Different McDonald Criteria

One hundred thirty-six of 325 patients (42%) were diagnosed with MS according to the current McDonald criteria of 2010 (Table 3). In the same cohort, only 70 of 325 patients (22%) would have been diagnosed with MS when the old McDonald criteria of 2005 or 2001 were applied. As the McDonald criteria of 2001 and 2005 were similar regarding evidence of dissemination in space, we found the same numbers of patients with newly diagnosed MS in our cohort. The importance of spinal cord lesion was emphasized in the criteria of 2005, as a spinal-cord lesion can replace an infratentorial lesion and any number of spinal-cord lesions can be included in total lesion count. However, these additional criteria regarding dissemination in space did not change the numbers of newly diagnosed MS in our cohort.

Although almost twice as many patients were diagnosed with MS according to the current McDonald criteria of 2010 we found quite similar inflammatory CSF changes when compared to patients classified to the old McDonald criteria of 2005/2001 (Table 3). Pleocytosis was present in 65% of MS patients classified with the current criteria versus 66% of patients classified with the old criteria. The quantitative intrathecal synthesis of the immunoglobulins IgG, IgM, and IgA was similarly distributed (Table 3). Remarkably the eminent high prevalence of OCB established for the 2005 criteria (98.5%) remained high with 96% after the patients had been classified according to the McDonald criteria of 2010.

### 2.5. Subgroup Analysis of CSF of MS Patients Diagnosed according to the McDonald Criteria of 2010

As described above, 136 patients were diagnosed with MS according to the revised McDonald criteria of 2010. The first subgroup consisting of 60 patients presented with the first relapse and diagnosis was done due to dissemination in space and time demonstrated by MRI (Table 4). In this group, 43 patients (72%) exhibited an increased CSF cell count while all (100%) presented OCB restricted to the CSF. In the second subgroup 39 patients diagnosed with MS reported one or more previous relapses and MRI showed both dissemination in space and time. 22 of these patients (56%) exhibited an increased CSF cell count and all but one (97%) presented OCB. The third subgroup consisted of 37 patients that reported one or more previous relapses but MRI showed dissemination in space only. In this subgroup, increased CSF cell count was found in 24 patients (65%) and OCB were present in 33 patients (89%).

## 3. Discussion

This is the first study that analyzed the prevalence of OCB in patients diagnosed with MS according to the McDonald criteria of 2010 at first presentation. In our cohort we found that 96% of patients with newly diagnosed MS displayed CSF OCB. Whereas no comparable OCB data exist for the McDonald criteria of 2010, two recent meta-analyses provide pooled sensitivities of OCB for patients classified according to the McDonald criteria 2005/2001. Petzold and colleagues calculated a sensitivity of 93% (53%–100%) from 49 studies with 11,136 patients [16], while Dobson and colleagues arrived a rate of 87.7% for 12,253 patients with MS and 68.6% for 2685 patients with CIS [13]. Our own cohort employing the criteria of 2005/2001 comprised 70 patients and reached a sensitivity of 98.5%.

It has been claimed that the McDonald criteria revisions of 2010 in some instances will allow a more rapid diagnosis of MS with equivalent or improved specificity and/or sensitivity compared with the previous criteria and will clarify and simplify the diagnostic procedure with fewer MRI examinations [15]. Our data agree with these expectations. While only 70 of 325 patients (22%) could be designated as MS according to the McDonald criteria of 2005, the number of newly diagnosed MS almost doubled to 136 patients (42%) in the same cohort, when the new criteria of 2010 were applied. Most importantly this remarkable increase in clinical sensitivity reduced the prevalence of OCB only marginal which dropped slightly from 98.5% to 96%.

Considering the fact that more MS patients can be diagnosed in an early stage of the disease, the new criteria have achieved their goal. However, a word of caution might be indicated. While only one patient lacked OCB in the 70 patients classified as MS according to the McDonald criteria of 2005/2001, this number rose to 5 of 136 patients, when the new criteria of 2010 were applied. A conversion to OCB positivity has been described in follow-up CSF investigations in about 50% of MS patients with initial absence of OCB [17]. Furthermore, Davies and colleagues reported that 9 of 27 patients showing a monoclonal band in IEF at the first lumbar puncture developed a clear oligoclonal response over a 6-year follow-up period, indicating a propensity for an inflammatory autoimmune driven CNS disease [18]. In our opinion a second lumbar puncture would be desirable to identify patients who develop OCB in the course of disease: on the one hand to obtain additional evidence supporting the diagnosis MS and on the other hand to identify patients with negative OCB as patients without OCB are considered to have a benign prognosis [9,17]. Repeated MRI and re-examination of the CSF were already suggested by others for OCB negative MS patients [17]. The German Society for Neurology suggests a control lumbar puncture after one year. Nevertheless, a low number of clinically definitive MS patients remain persistently OCB negative [9,17].

Another important aspect is the prevalence of OCB in patients diagnosed with CIS. Since more patients were diagnosed with MS according to the latest McDonald criteria the group of patients diagnosed with CIS decreased substantially from 255 patients (McDonald 2005) to 189 patients (McDonald 2010). As expected the prevalence of OCB in the 189 CIS patients was lower with 55% when compared to patients diagnosed with CIS according to the previous McDonald criteria of 2005/2001 with 65%, which compares well to the 68.6% in the meta-analysis of Dobson and colleagues [13].

Previous studies have shown that the detection of OCB is considered to be a risk factor for developing MS. The crucial role of OCB was shown by the work of Tintore and colleagues as 23% (7/30) of CIS patients with OCB but without MRI lesions developed MS in contrast to only 4% (3/83) of CIS patients without OCB [19]. A recent meta-analysis by Kuhle and colleagues identified that the presence of OCB in CIS patients duplicates the risk to develop MS independent of MRI findings [10]. The lack of MRI lesions and OCB represents a low risk, while the detection of both factors means high risk.

CSF OCB represent a humoral immune reaction in the CNS, and are thus, methodologically in contrast to MRI in which lesions are detected morphologically. Both methods complement each other. The role of B lymphocytes comes into the focus of MS research and therapeutic approaches target B cells. Even more important is the fact that the phase III trial with the monoclonal antibody ocrelizumab which targets CD20 positive B cells not only has shown beneficial effects on relapsing MS but also on primary progressive MS with positive OCB.

In our cohort CIS patients with spinal cord lesions and brainstem lesions showed a higher prevalence of OCB than patients with optic neuritis. The relevance of lesion location in CIS patients as a risk factor for conversion to MS is controversially discussed. Clinical trials performed on CIS patients did not show differences between different topographies of lesions. However, Tintore and colleagues identified patients with optic neuritis to have a lower risk for a second relapse [20]. They found that CIS patients with optic neuritis showed a lower prevalence of OCB which is similar to our results. One possible reason for this observation might be that patients with visual problems not associated with demyelinating disease have been diagnosed as optic neuritis. In contrast to optic neuritis, brainstem and spinal lesions are rather more specific to be the result of a demyelinating disease. However, long-term surveillance of CIS patients is necessary to further clarify this aspect.

In conclusion, our results show that the prevalence of OCB remains high when patients are diagnosed with MS according to the McDonald criteria of 2010. Although CSF analysis is not mandatory for diagnosis of RRMS according to the McDonald criteria of 2010, the evidence of an intrathecal humoral immunoreaction is a useful additional information in the diagnostic workup. Lumbar puncture with detection of OCB should be performed in all patients with MS and CIS at first diagnosis and a negative OCB status should lead to a critical reevaluation of the diagnosis.

## 4. Methods

### 4.1. Patients

In this retrospective study we screened the medical records of all patients who were admitted to the Department of Neurology of the Hannover Medical School with relapse symptoms suggestive for MS in the time from 2010 to 2015. Patients that have been already diagnosed with MS were not included. Demographic, clinical, laboratory and CSF data were acquired. MRI of the brain and CSF were available in all patients. Standardized laboratory testing (antinuclear antibodies, anti-DNA antibodies, antiphospholipid antibodies, antineutrophil cytoplasmic antibodies, HIV, borrelia burgdorferi antibodies, antibodies to Treponema pallidum) was performed to exclude other autoimmune causes such as connective tissue diseases or infectious diseases that could mimic MS. Patients were divided into two groups, MS or CIS, according to the McDonald criteria 2010. To compare the CSF results with previous criteria, in the second part, the same patients were divided into the groups MS or CIS according to the McDonald criteria 2005 and 2001. This investigation was approved by the institutional ethics committee of the Hannover Medical School (No. 3033-2016, 27 January 2016). This is a retrospective study and only data were included that were evaluated for patients treatment.

### 4.2. CSF and Serum Analytical Procedures

CSF and serum were analysed by routine methods in the neurochemistry laboratory of the Department of Neurology [21]. CSF cells were counted manually with a Fuchs-Rosenthal counting chamber. CSF total protein (cut-off = 500 mg/L) was determined by a Bradford dye-binding procedure. IgG, IgA, IgM, and albumin were measured in CSF and serum in the same latex enhanced assay by kinetic nephelometry (Beckman Coulter IMMAGE). Blood-CSF barrier function was estimated as CSF-serum albumin quotients (QAlb). The age-adjusted upper reference limit of QAlb was calculated using the formula QAlb = 4 + (age in years/15) [22]. Intrathecal synthesis of IgG, IgA, and IgM was calculated according to the method of Reiber-Felgenhauer referring the IgG, IgA, and IgM quotients to QAlb [22]. CSF-specific OCB were determined by isoelectric focusing in polyacrylamide gels with consecutive silver staining. Five patterns of OCB were distinguished, following the recommendations of the first European consensus on CSF analysis in MS [23]. Two bands restricted to the CSF only were sufficient for a positive rating, but weak type 2 or type 3 patterns with only 2–3 bands were separately registered as type 2a or type 3a. For all protein analyses, CSF and serum samples were analysed within the same analytical series. All methods are quality assured by participating in external quality control programs, the CSF survey of INSTAND [24].

### 4.3. Magnetic Resonance Imaging (MRI)

All patients were examined by cerebral MRI at baseline and, when available, spinal MRI was additionally taken into analysis. All MRI examinations of the brain included a T1-weighted, T2-weighted, and gadolinium-enhanced T1-weighted sequence. The number of T2-weighted lesions and the presence of gadolinium-enhanced lesions were evaluated according to the following MR criteria for MS: Swanton criteria of 2006 for diagnosis according to the McDonald Criteria of 2010 [25], Barkhof criteria of 1997 for diagnosis according to the McDonald criteria of 2000 and Tintore-Barkhof criteria of 2000 according to the McDonald criteria of 2005 [26].

### 4.4. Statistical Analysis

GraphPad Prism version 5.02 was used for statistical analysis. The D’Agostino–Pearson normality test was used to prove whether values were normally distributed. The two-tailed unpaired two-sided Mann-Whitney test was performed for comparison of two independent groups and the Fisher’s exact test was used to assess the statistical significance in categorical data. The level of statistical significance was set to 5%. Data was described by medians and interquartile ranges.

## Figures and Tables

**Table 1:** Cerebrospinal fluid results.

Characteristics (McDonald 2010)	Patient Numbers	Pleocytosis (≥5 cells/µL)	Lactate (>3.5 mmol/L)	Protein (>500/mg/L)	Blood-CSF-Barrier Dysfunction	Intrathecal Synthesis			CSF Oligoclonal Bands
						IgM	IgG	IgA	
**Multiple Sclerosis**	**136**	**65%**	**1%**	**28%**	**26%**	**35%**	**63%**	**13%**	**96%**
Optic neuritis	44	66%	0%	25%	18%	32%	64%	11%	98%
Paresis/Sensory symptoms	30	63%	0%	27%	30%	30%	73%	20%	97%
Brainstem symptoms	19	79%	0%	26%	21%	37%	47%	16%	95%
Spinal cord symptoms	30	57%	0%	33%	37%	40%	57%	7%	93%
Polysymptomatic	13	69%	8%	31%	31%	46%	69%	15%	100%
**Clinically Isolated Syndrome**	**189**	**52%**	**0%**	**21%**	**22%**	**14%**	**33%**	**4%**	**55%**
Optic neuritis	136	50%	0%	18%	23%	13%	23%	3%	46%
Paresis/Sensory symptoms	11	45%	0%	27%	9%	27%	45%	27%	55%
Brainstem symptoms	16	44%	0%	19%	13%	19%	63%	0%	88%
Spinal cord symptoms	26	69%	0%	35%	31%	15%	62%	4%	85%
*p* value	-	*p* = 0.1	*p* = 1.0	*p* = 0.3	*p* = 0.6	*p* = 0.0009	*p* = 0.0001	*p* = 0.04	*p* = 0.0001

Cerebrospinal fluid laboratory findings in patients diagnosed with multiple sclerosis and clinically isolated syndrome according to the McDonald criteria 2010; *p* values indicate comparison between multiple sclerosis and clinically isolated syndrome.

**Table 2:** Different combinations of intrathecal synthesis of immunoglobulins IgG, IgM, and IgA.

Combinations of Antibody Classes	Multiple Sclerosis		Clinically Isolated Syndrome	
	Intrathecal Synthesis of Immunoglobulins			
	Reiber-Felgenhauer	Reiber-Felgenhauer + OCB	Reiber-Felgenhauer	Reiber-Felgenhauer + OCB
3-class synthesis of IgG + IgM + IgA	7%	8%	3%	3%
2-class synthesis of IgG + IgM	21%	27%	11%	11%
2-class synthesis of IgG + IgA	4%	5%	1%	1%
2-class synthesis of IgM + IgA	1%	0	1%	0
Isolated synthesis of IgG	29%	54%	19%	41%
Isolated synthesis of IgM	5%	0	1%	0
Isolated synthesis of IgA	1%	0	0	0

Presentation of different combinations of intrathecal synthesis of immunoglobulins IgG, IgM, and IgA; data show values assessed by the method of Reiber-Felgenhauer (quantitative method) and in combination with oligoclonal bands (OCB; qualitative method of intrathecal IgG synthesis).

**Table 3:** The McDonald criteria compared.

Classification	McDonald Criteria	McDonald Criteria	Pleocytosis (≥5 cells/µL)	Intrathecal Synthesis			CSF Oligoclonal Bands
				IgM	IgG	IgA	
Multiple sclerosis	2005/2001	70	66%	37%	64%	16%	98.5%
	2010	136	65%	35%	63%	13%	96%
*p* value	-	*p* = 0.0001	*p* = 1.0	*p* = 0.9	*p* = 1.0	*p* = 0.7	*p* = 0.4
Clinically isolated syndrome	2005/2001	255	55%	19%	40%	6%	65%
	2010	189	52%	14%	33%	4%	55%
*p* value	-	*p* = 0.0001	*p* = 0.8	*p* = 0.4	*p* = 0.4	*p* = 0.7	*p* = 0.2

Cerebrospinal fluid findings in patients with multiple sclerosis and clinically isolated syndrome according to the McDonald criteria 2010 in comparison to the McDonald criteria 2005/2001.

**Table 4:** Multiple sclerosis subgroups compared.

Multiple Sclerosis (McDonald 2010) Subgroups	Patient Numbers	Pleocytosis (≥5 cells/µL)	Intrathecal Synthesis			CSF Oligoclonal Bands
			IgM	IgG	IgA	
1 relapse and MRI dissemination in space and time	60	72%	35%	65%	12%	100%
≥2 relapses and MRI dissemination in space and time	39	56%	36%	59%	15%	97%
≥2 relapses and MRI dissemination in space only	37	65%	32%	62%	14%	89%

Comparison of cerebrospinal fluid findings in three subgroups of patients with multiple sclerosis diagnosed according to the McDonald criteria 2010.

## References

[1] Miller D.H., Chard D.T., Ciccarelli O. (2012). Clinically isolated syndromes. Lancet Neurol..

[2] Compston A., Coles A. (2002). Multiple sclerosis. Lancet.

[3] Comi G., Filippi M., Barkhof F., Durelli L., Edan G., Fernandez O., Hartung H.P., Seeldrayers P., Sørensen P.S., Rovaris M. (2001). Effect of early interferon treatment on conversion to definite multiple sclerosis: A randomised study. Lancet.

[4] Comi G., Martinelli V., Rodegher M., Moiola L., Bajenaru O., Carra A., Elovaara I., Fazekas F., Hartung H.P., Hillert J. (2009). Effect of glatiramer acetate on conversion to clinically definite multiple sclerosis in patients with clinically isolated syndrome (PreCISe study): A randomised, double-blind, placebo-controlled trial. Lancet.

[5] Jacobs L.D., Beck R.W., Simon J.H., Kinkel R.P., Brownscheidle C.M., Murray T.J., Simonian N.A., Slasor P.J., Sandrock A.W. (2000). Intramuscular interferon β-1a therapy initiated during a first demyelinating event in multiple sclerosis. N. Engl. J. Med..

[6] Kappos L., Polman C.H., Freedman M.S., Edan G., Hartung H.P., Miller D.H., Montalban X., Barkhof F., Bauer L., Jakobs P. (2006). Treatment with interferon β-1b delays conversion to clinically definite and McDonald MS in patients with clinically isolated syndromes. Neurology.

[7] Stangel M., Fredrikson S., Meinl E., Petzold A., Stuve O., Tumani H. (2013). The utility of cerebrospinal fluid analysis in patients with multiple sclerosis. Nat. Rev. Neurol..

[8] Tumani H., Hartung H.P., Hemmer B., Teunissen C., Deisenhammer F., Giovannoni G., Zettl U.K., BioMS Study Group (2009). Cerebrospinal fluid biomarkers in multiple sclerosis. Neurobiol. Dis..

[9] Link H., Huang Y.M. (2006). Oligoclonal bands in multiple sclerosis cerebrospinal fluid: An update on methodology and clinical usefulness. J. Neuroimmunol..

[10] Kuhle J., Disanto G., Dobson R., Adiutori R., Bianchi L., Topping J., Bestwick J.P., Meier U.C., Marta M., Dalla Costa G. (2015). Conversion from clinically isolated syndrome to multiple sclerosis: A large multicentre study. Mult. Scler..

[11] Bosca I., Magraner M.J., Coret F., Alvarez-Cermeno J.C., Simo-Castello M., Villar L.M., Casanova B. (2010). The risk of relapse after a clinically isolated syndrome is related to the pattern of oligoclonal bands. J. Neuroimmunol..

[12] Joseph F.G., Hirst C.L., Pickersgill T.P., Ben-Shlomo Y., Robertson N.P., Scolding N.J. (2009). CSF oligoclonal band status informs prognosis in multiple sclerosis: A case control study of 100 patients. J. Neurol. Neurosurg. Psychiatry.

[13] Dobson R., Ramagopalan S., Davis A., Giovannoni G. (2013). Cerebrospinal fluid oligoclonal bands in multiple sclerosis and clinically isolated syndromes: A meta-analysis of prevalence, prognosis and effect of latitude. J. Neurol. Neurosurg. Psychiatry.

[14] Freedman M.S., Thompson E.J., Deisenhammer F., Giovannoni G., Grimsley G., Keir G., Öhman S., Racke M.K., Sharief M., Sindic C.J. (2005). Recommended standard of cerebrospinal fluid analysis in the diagnosis of multiple sclerosis: A consensus statement. Arch. Neurol..

[15] Polman C.H., Reingold S.C., Banwell B., Clanet M., Cohen J.A., Filippi M., Fujihara K., Havrdova E., Hutchinson M., Kappos L. (2011). Diagnostic criteria for multiple sclerosis: 2010 revisions to the McDonald criteria. Ann. Neurol..

[16] Petzold A. (2013). Intrathecal oligoclonal IgG synthesis in multiple sclerosis. J. Neuroimmunol..

[17] Zeman A.Z., Kidd D., McLean B.N., Kelly M.A., Francis D.A., Miller D.H., Kendall B.E., Rudge P., Thompson E.J., McDonald W.I. (1996). A study of oligoclonal band negative multiple sclerosis. J. Neurol. Neurosurg. Psychiatry.

[18] Davies G., Keir G., Thompson E.J., Giovannoni G. (2003). The clinical significance of an intrathecal monoclonal immunoglobulin band: A follow-up study. Neurology.

[19] Tintore M., Rovira A., Rio J., Tur C., Pelayo R., Nos C., Tellez N., Perkal H., Comabella M., Sastre-Garriga J. (2008). Do oligoclonal bands add information to MRI in first attacks of multiple sclerosis?. Neurology.

[20] Tintore M., Rovira A., Rio J., Otero-Romero S., Arrambide G., Tur C., Comabella M., Nos C., Arévalo M.J., Negrotto L. (2015). Defining high, medium and low impact prognostic factors for developing multiple sclerosis. Brain.

[21] Skripuletz T., Schwenkenbecher P., Pars K., Stoll M., Conzen J., Bolat S., Pul R., Vonberg R.P., Sedlacek L., Wurster U. (2014). Importance of follow-up cerebrospinal fluid analysis in cryptococcal meningoencephalitis. Dis. Markers.

[22] Reiber H. (1998). Cerebrospinal fluid-physiology, analysis and interpretation of protein patterns for diagnosis of neurological diseases. Mult. Scler..

[23] Andersson M., Alvarez-Cermeno J., Bernardi G., Cogato I., Fredman P., Frederiksen J., Fredrikson S., Gallo P., Grimaldi L.M., Grønning M. (1994). Cerebrospinal fluid in the diagnosis of multiple sclerosis: A consensus report. J. Neurol. Neurosurg. Psychiatry.

[24] Reiber H. (1995). External quality assessment in clinical neurochemistry: Survey of analysis for cerebrospinal fluid (CSF) proteins based on CSF/serum quotients. Clin. Chem..

[25] Swanton J.K., Rovira A., Tintore M., Altmann D.R., Barkhof F., Filippi M., Huerga E., Miszkiel K.A., Plant G.T., Polman C. (2007). MRI criteria for multiple sclerosis in patients presenting with clinically isolated syndromes: A multicentre retrospective study. Lancet Neurol..

[26] Tintore M., Rovira A., Martinez M.J., Rio J., Diaz-Villoslada P., Brieva L., Borrás C., Grivé E., Capellades J., Montalban X. (2000). Isolated demyelinating syndromes: Comparison of different MR imaging criteria to predict conversion to clinically definite multiple sclerosis. Am. J. Neuroradiol..

